# Synthesis, Spectroscopic, Thermodynamics and Kinetics Analysis Study of Novel Polymers Containing Various Azo Chromophore

**DOI:** 10.1038/s41598-019-57264-3

**Published:** 2020-01-16

**Authors:** Dilek Çanakçı

**Affiliations:** Department of Chemistry, Vocational School of Technical Sciences, Adıyaman University, 02040, Adıyaman, Turkey

**Keywords:** Polymer synthesis, Solution-state NMR

## Abstract

Two novel polymers containing azo and ether groups were synthesized by oxidative polycondensation in an aqueous alkaline medium by NaOCI oxidants. The azo dye monomers that were polymerized were synthesized by diazotization of 2-amino-4-chlorophenyl phenyl ether and coupling reaction with 2,7-dihydroxynaphthalene and 1,3-benzenediol. Structures of the synthesized compounds were characterized by spectroscopic studies such as FT-IR, UV-vis, ^1^H-NMR. Gel permeation chromatography was used to evaluate the molecular weight and molecular weight distribution of the azo polymers. Furthermore, the surface morphology of the azo monomers and polymers were scrutinized by using scanning electron microscope. To investigate the effect of solvent on absorption, the electronic absorption spectra of the synthesized compounds were measured in six solvents with different polarity. The thermal behaviors of the monomers and polymers were identified by the TG, DTG and DTA techniques. In addition, the Coats-Redfern, Horowitz-Metzger and Broido methods for the determination of the kinetic parameters were used in the kinetic analysis of thermal decomposition of the compounds.

## Introduction

Polymer is a molecule of high relative molecular mass, the structure of which essentially comprises the multiple repetition of units derived, actually or conceptually, from molecules of low relative molecular mass. As polymers are the substances that have adequate mechanical features, can be shaped easily, and are chemical resistant; they are used in the manufacturing of plastics, rubber, fibers, dyes, and adhesive type materials, which are used widely in every area of our daily lives. In order for the monomers, which have small molecular weights, to form polymer chains, they should include two or more bonds. The method to be used for polymerization also varies according to the type of monomers. One of the methods used in forming polymers is condensation polymerization. In order for this method to occur, the monomer that will form the polymer should be bifunctional. A small molecule such as H_2_O, HCI is eliminated from the structure during the condensation polymerization. The existence of different groups in the structure of polymers causes changes in their physical and chemical properties. Today, there are many natural and synthetic dyestuffs that are used to get differently colored materials. Dyestuff is classified according to the type of chromophore group on the dye molecule. Azo dyes contain one or more azo groups (N=N) having aromatic rings. They are the oldest and largest class of industrial synthesized organic dyes^1–3^. Over the past few decades, azobenzene-based dyes have been extensively studied in the field of material chemistry as well as dye industry^4–7^. Their most common use is in the textile industry, especially wool, leather and synthetic fabrics due to their excellent coloring properties^8^. They are also employed in the food^9^, cosmetic^10^, printing^11^, and pharmaceutical industries^12^, in colored plastics^13^ and polymers^14^. However, they are applied in many other areas owing to their versatility such as biomedical studies, advanced application in organic synthesis and technical fields such as laser, liquid crystalline displays and inkjet printers^15–20^. Furthermore, the azobenzene derivatives affect the optical properties of polymer materials containing them^21–25^, due to the reversible E → Z → E photoisomerization cycle, light-induced dichroismand optical birefringence^26^. This matchless molecular optical switching of azobenzene derivatives creates photorefractive effects^27–36^ used in holographic recording and optical poling grating.

In the present work, the synthesis of a novel naphthol and phenol based azo dyes and the polymerization of obtained azo dyes by the oxidative polycondensation reaction in an aqueous alkaline medium was reported. The average molecular weights of synthesized polymers were determined by gel permeation chromatography (GPC). FT-IR and ^1^H-NMR spectra were obtained to determine the structure of the azo monomers and polymers. The morphology of the produced monomers and polymers has been investigated by means of scanning electron microscopy (SEM). Also, thermal stabilities of synthesized compounds were studied by TG, DTG, DTA techniques. Further, the effects of solvents on the absorption spectra of the synthesized azo monomers and polymers have been investigated.

## Experiment

### Materials and instrumentation

All chemicals and solvents used for the synthesis were purchased from Sigma Aldrich Chemical Company and used without further purification. Molecular weights of the polymer were determined by Shimadzu LC Solution GPC equipped with Shodex 80 M and K-802 column and RID-6A refractive index detector at 40 °C. Also, kloroform was used as the eluent at flow rate of 0.8 mL/min. FT-IR spectra were performed on a Perkin Elmer-Spectrum 100 FT-IR instrument as KBr pellets in the region of 4000-650 cm^−1^. By using Perkin Elmer Lambda 35 ES UV/Vis Spectrophotometer, the UV-visible spectra of the all synthesized compounds were measured in the range of 200–700 nm in different solvents, such as DMSO, Dichloromethane(DMC), acetone, ethanol, dioxane, acetic acid at concentrations (3 × 10^−5^ mol/L). ^1^H-NMR spectra were performed by using a Bruker 300 MHz spectrometer at room temperature in CDCl_3_ as a solvent and with tetramethylsilane (TMS) as an internal standard. Chemical shifts were reported in parts permillion (ppm). By using Hitachi7300 thermal analyzer, the thermal analysis (TG-DTA) was recorded between 30 and 700 °C under the N_2_ atmosphere. In the study, modifications of the surface structure of monomers and polymers were determined by Zeiss EVO LS 10 Scanning Electron Microscope (SEM).

### Synthesis of azo monomer

#### Synthesis of azo monomer M_1_

**Figure 1:**
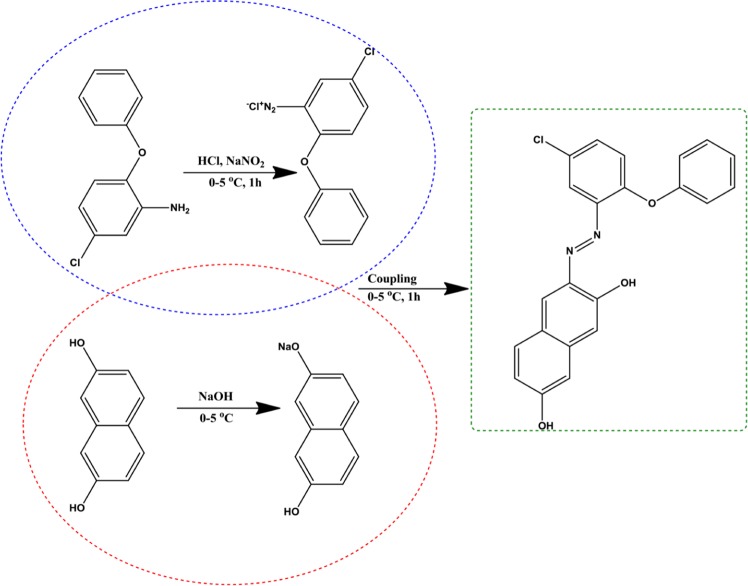
The synthetic route of azo monomer M_1_.

2-Amino-4-chlorophenyl phenyl ether (4.38 g, 0.02 mol) was added into the mixture of HCl (0.06 mol, 5.6 mL of 30% HCl) and water (2 mL). The aromatic amine solution was stirred until a clear solution was obtained. Then it was cooled to 0–5 °C. The solution of sodium nitrite (0.138 g, 0.02 mol, 5 ml) was dissolved in an ice bath and added dropwise to the reaction mixture over 45 min. during the stirring process. After the completion of diazotization, the cooled basic solution of 2,7-dihydroxynaphthalene (3.2 g, 0.02 mol) in sodium hydroxide solution (0.8 g, 0.02 mol, 0.8 mL, 0–5 °C) was added dropwise to the prepared diazonium solution and the mixture was stirred for 20 min. The precipitate was filtered and dried after washing with cold water. The crude product (3-((5-chloro-2-phenoxyphenyl)diazenyl)naphthalene-2,7-diol) was obtained and recrystallized from ethanol/water (1/5) (Fig. 1). The yield was 95% as claret red powder. FT-IR (KBr, cm^−1^): 3060(O-H), 2922-2734 (C-H, aromatic), 1618-1537 (C=C, aromatic), 1475 (N=N). UV-visible (DMSO, nm): 230, 309, 483. ^1^H NMR (300 MHz, CDCl_3_) δ: 15.90 (s, -OH), 12.39 (s, -OH), 9.19 (s, 1 H). 7.94 (d, J = 43.8 Hz, 1 H), 7.89 (s, 1 H), 7.82 (d, J = 59.3 Hz, 1 H), 7.66 (s, 1 H), 7.59–6.84 (m, 3 H), 7.02 (d, J = 38.5 Hz, 1 H), 7.02 (d, J = 38.5 Hz, 1 H), 6.89 (d, J = 36.4 Hz, 1 H), 6.83 (s, 1 H), 6.72 (d, J = 8.3 Hz, 1 H), 6.55 (d, J = 10.1 Hz, 1 H).

#### Synthesis of azo monomer M_2_

This compound was prepared as described in previous method by using 2-amino-4-chlorophenyl phenyl ether and 1,3-benzenediol. Obtained compound (4-((5-chloro-2-phenoxyphenyl)diazenyl) benzene-1,3-diol) was recrystallized twice from ethanol/water as red powder: yield 90%. FT-IR (KBr, cm^−1^): 3068 (O-H), 2645 (C-H, aromatic), 1613-1586 (C=C, aromatic), 1464 (N=N). UV-visible (DMSO, nm): 240, 416. ^1^H NMR (300 MHz, CDCl_3_) δ: 15.48 (s, -OH), 8.07 (s, 1 H), 7.89 (s, 1 H), 7.64 (d, *J* = 9.0 Hz, 1 H), 7.59 (d, *J* = 9.5 Hz, 1 H), 7.48-7.38 (m, 2 H), 7.27 (d, *J* = 15.4 Hz, 1 H), 7.14 (d, *J* = 7.5 Hz, 1 H), 7.04 (d, *J* = 8.8 Hz, 1 H), 6.92 (d, *J* = 6.7 Hz, 1 H), 6.58 (d, *J* = 9.4 Hz, 1 H), 5.50 (s, -OH).

### Synthesis of azo polymer

#### Synthesis of azo polymer P_1_

**Figure 2:**
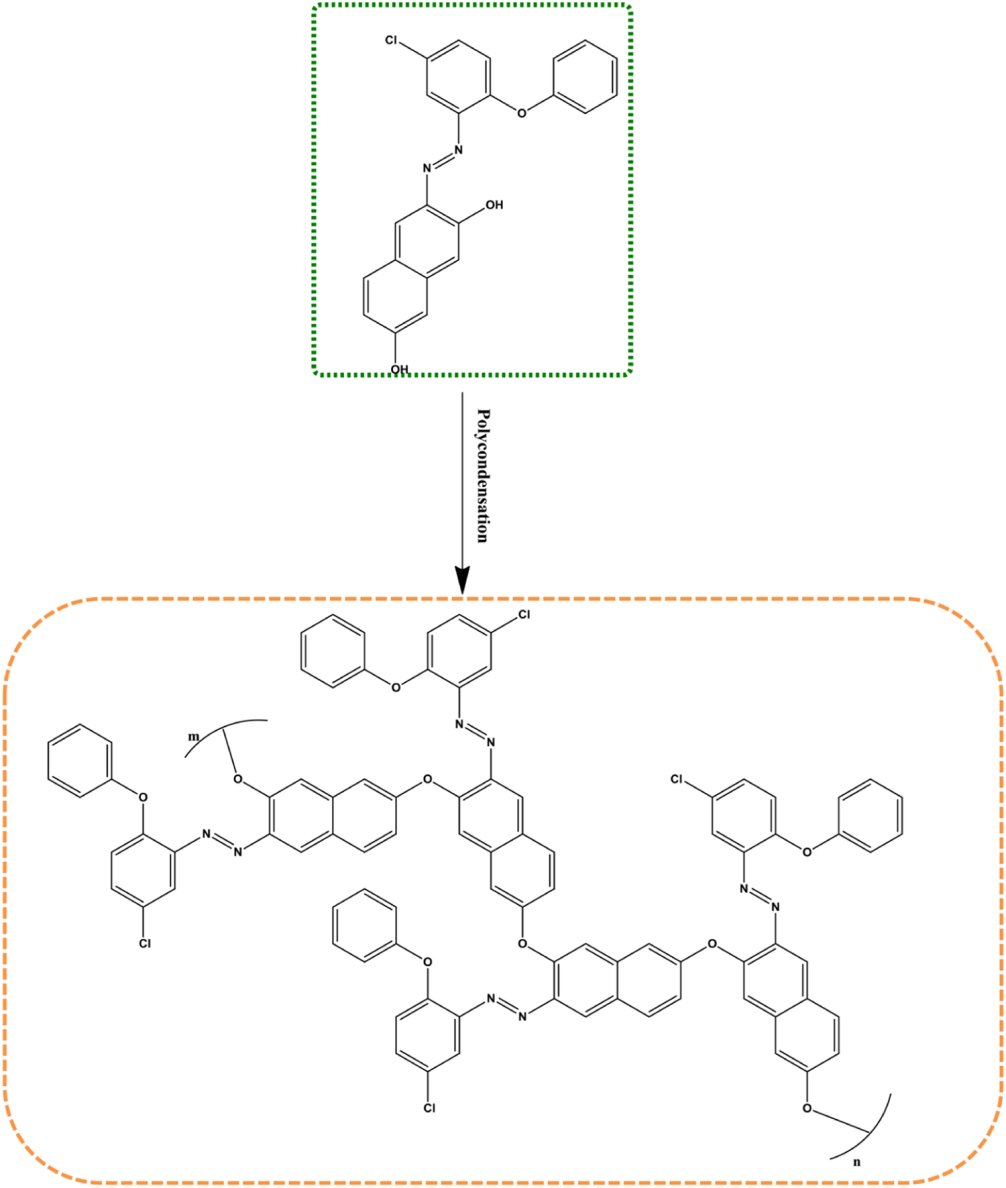
The synthetic route of azo polymer P_1_.

The polymer P_1_ was synthesized by using oxidative polycondensation method. After azo monomer M_1_ (39 g, 0.01 mol) was placed in the flask, an aqueous solution of KOH (10%, 0.04 mol) was added over it. The solution was heated for 30 min at 70 °C. At the end of the 30 min., NaOCl (0.6 ml, 0.06 mol) was added dropwise into the hot mixture over about 1 h. After the oxidant insertion process, the temperature of the mixture was increased to 95 °C and stirred at this temperature for 8 h. HCl (37%, 0,5 ml, 0.04 mol) was used to terminate the polymerization at the end of the period. Then, the obtained polymer was washed with benzene (5 ml) in order to remove any residual monomer, filtered and dried in an oven at 105 °C (Fig. 2). The obtained compound (poly-(3-((5-chloro-2-phenoxyphenyl)diazenyl)naphthalene-2,7-diol) was purified twice from ethanol/water. The yield was 85% as black powder. FT-IR (KBr, cm^−1^): 3245 (O-H), 3064(C-H, aromatic), 1712-1543 (C=C, aromatic), 1490-1455 (N=N), 1216-1019 (C-O-C). UV-visible (DMSO, nm): 217, 240, 308, 484. ^1^H NMR (300 MHz, CDCl_3_) δ: 15.63 (s, -OH), 15.36 (s, -OH), 14.57 (s, -OH), 8.57-5.64 (m, Ar-H).

#### Synthesis of azo polymer P_2_

This compound was prepared as described in previous method by using M_2_. Obtained compound (poly-(4-((5-chloro-2-phenoxyphenyl)diazenyl) benzene-1,3-diol)) was purified twice from ethanol/water as black powder: yield 90%. FT-IR (KBr, cm^−1^): 3066 (-OH and C-H, aromatic), 1611-1586 (C=C, aromatic), 1513-1407 (N=N), 1280-1023 (C-O-C). UV-visible (DMSO, nm): 247, 292, 451. ^1^H NMR (300 MHz, CDCl_3_) δ: 15.69 – 15.57 (m, -OH), 15.41 (s, -OH), 15.44 (s, -OH), 14.52 (m, -OH), 8.12 (d, J = 18.1 Hz, 93 H), 7.89 (m, 97 H), 7.86 (d, J = 22.9 Hz, 111 H), 7.40-7.26 (m, 385 H), 7.22 (s, 193 H), 7.19-6.84 (m, 992 H), 7.60-4.65 (m, 231 H), 7.40-4.65 (m, 197 H), 6.84-5.41 (m, 376 H), 6.33 (d, J = 10.1 Hz, 94 H).

## Results and Discussion

### FT-IR analysis

**Figure 3:**
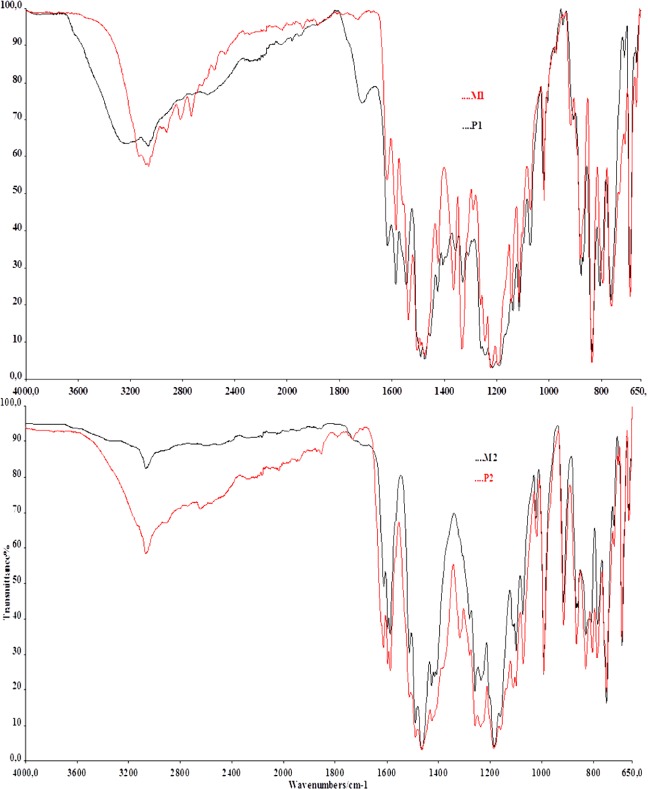
The FT-IR spectrum of azo monomers M_1_, M_2_ and polymers P_1_, P_2_.

The chemical structures of synthesized compounds were confirmed by FT-IR spectroscopic method. The FT-IR spectra of the azo monomers and polymers were recorded as KBr pellets in the region 4000-650 cm^−1^ (Fig. 3). The infrared spectra of M_1_ under the study showed a weak band at 3068 cm^−1^ which corresponded to the -OH of the non-hydrogen-bonded groups and the aromatic C-H stretching vibration occured in the range 2922-2734 cm^−1^. The aromatic C-H in-plane bending and out-of-plane bending vibration appeared in 1332-1101 cm^−1^ and 1070-650 cm^−1^, respectively. While the bands in 1618-1537 cm^−1^ region corresponded to the aromatic C=C stretching vibration of aromatic rings, the other band in the 1475 cm^−1^ region was formed as a result of the N=N stretching vibration of azo group. The M_2_ showed a weak broad band around 3068 cm^−1^ due to aromatic -OH group. The band at 2645 cm^−1^ was due to the aromatic C-H vibration of M_2_. The bands observed in the range 1613-1589 in the FT-IR spectrum of the M_2_ was ascribed to the aromatic C=C stretching vibration. Also, the weak band observed at 1464 cm^−1^ was due to the presence of N=N group.

The FT-IR peaks of polymers were broader than monomers because their polyconjugated structures had been formed by polymerization. Also, the number of peaks in the spectrum decreased due to their conjugated systems. The most obvious difference was observed on the -OH stretching in the FT-IR spectra of the P_1_. While the O-H stretching of the M_1_ was seen as a small peak band, the -OH stretching band was observed as a widespread band at the spectra of the P_1_. The aromatic -OH and C-H stretching of the polymer was observed at 3245 and 3064 cm^−1^. The bands of aromatic C=C and N=N stretching vibration occurred in the range of 1712-1543 cm^−1^ and 1490-1455 cm^−1^, respectively. The peaks observed in the range of 1216-1019 cm^−1^ were due to C-O-C stretching vibrations of phenylene and oxyphenylene units in the polymers^37^. In the FT-IR spectra of the azo polymer P_2_, the broad absorption band appeared at 3066 cm^−1^ assigned to phenolic -OH group. The aromatic C-H stretching vibrations were not observed due to wide -OH band and the intensity band at 1611-1586 cm^−1^ assigned aromatic C=C stretching. Also, N=N group stretching bands of the P_2_ were observed in the range of 1513-1407 cm^−1^.

### ^1^H NMR analysis

**Figure 4:**
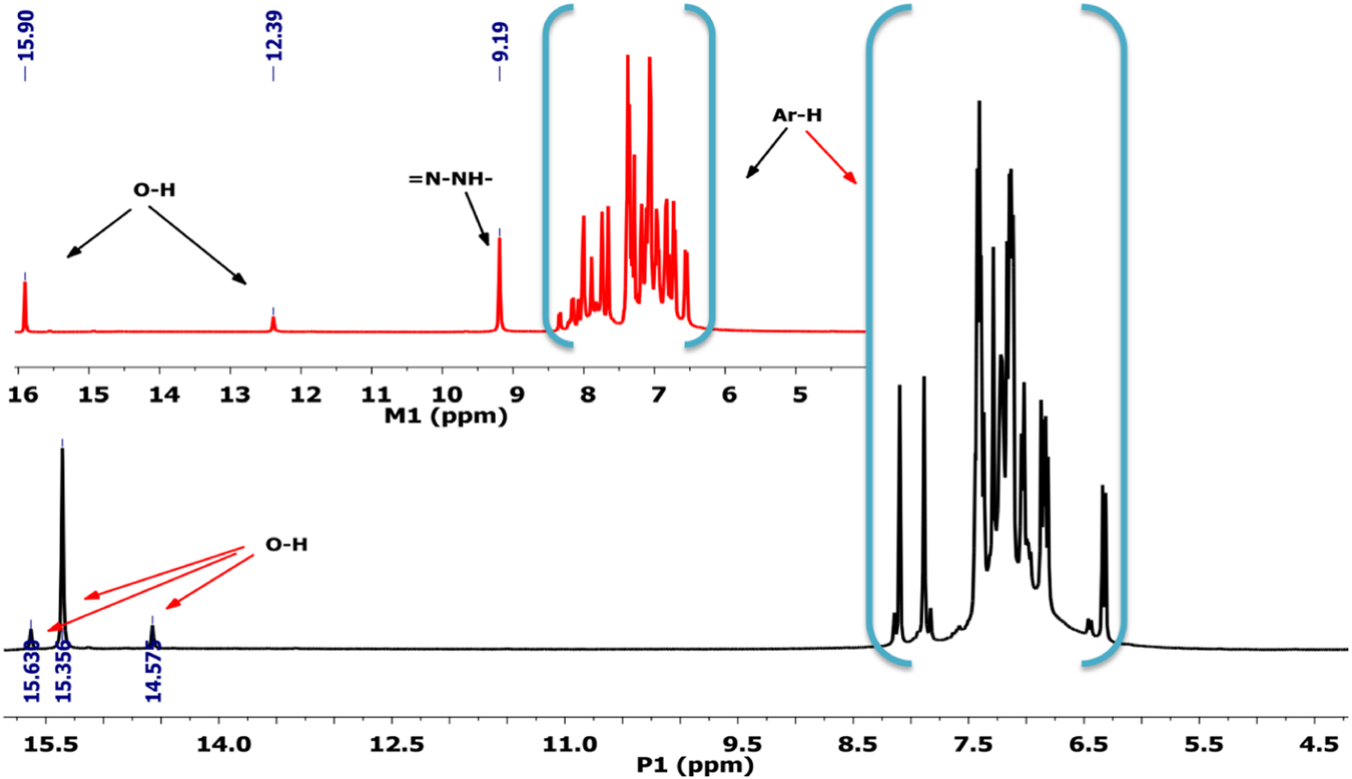
1HNMR spectrum of azo monomer M_1_ and polymer P_1_.

The chemical shift values at ^1^H-NMR spectra of synthesized azo monomer M_1_ and polymer P_1_ are given in Fig. 4. The signal of hydroxyl protons was observed in 15.90 and 12.39 ppm. The chemical shift of one of the hydroxyl hydrogens was greater than the others because of the formation of the intramolecular hydrogen bond with the nitrogen atom of N=N group. The singlet peak, which was assigned to the hydrazone proton (=N-NH-), was at 9.19 ppm. The protons on the aromatic ring of monomer were observed in the range of 6.55 and 7.94 ppm. The most distinctive difference supporting the polymer formation in the polymer spectrum was that the signals of the aromatic hydrogen were observed as a hill. The aromatic protons of polymer were observed in the range of 5.64-8.57 ppm as a multiplet. Because polymerization occurred via coupling at the ortho and para position of (C-O-C) oxyphenylene and aromatic ring, the -OH proton signal observed at 12.39 ppm in the monomer spectra which could not be observed in the spectra of polymer^38^. The signal of hydroxyl protons that were not included in the polymerization was observed at 15.63, 15.36 and 14.57 ppm.

In ^1^H NMR spectrum of M_2_, the characteristic signal of phenols protons was observed in the region 7.89-6.58 ppm. The singlet peak observed at 8.07 ppm was assigned to the hydrazone proton.The sharp weak peak at 15.48 ppm is due to hydrogen bonded phenolic hydroxyl proton. Since the other hydroxyl proton of phenol did not form a hydrogen bond, its signal was observed at 5.50 ppm. The multiplet peaks at 8.12-6.33 ppm correspond to aromatic protons in 1 H NMR spectrum of P_2_.The hydroxyl proton peak observed at 5.50 ppm in the M_2_ spectrum did not occur in the P_2_ spectrum due to its incorporation into the polymer formation. The other hydroxyl proton peak which did not participate in the polymer formation was observed in the region 15.69-14.52 ppm.

### GPC analysis

According to the GPC chromatograms, the calculated mass average number of molecular weight (Mn), the mass average weight of molecular weight (Mw), and polydispersity index (PDI) values of P_1_ were found as 11.140 × 10^2^ g mol^−1^, 11.900 × 10^2^ g mol^−1^ and 1,044, respectively. Deviation of polydispersity index value from 1 shows that the polymer occurred as a polymer chain with different molecular lengths. In light of this information, it was seen that the P_1_ had a narrow molecular distribution. The values obtained from the GPC chromatogram of P_2_ were Mn: 4.003 g mol^−1^ and Mw: 25.019 g mol^−1^ and PDI: 6,249.The results showed that P_2_ had a wide molecular distribution different from P_1_. Molecular weight distribution in polymers increases as polydispersity increases. The reason why P_1_ polymer has a narrow molecular weight distribution is that the polymer chain lengths are close to each other. This is because all the active centers appeared at the beginning of the polymerization because the reaction solution was mixed rapidly enough and homogeneous. In addition, all polymer chains have grown simultaneously because the growth rate and onset rate are competitive. As a result the reaction conditions led to the M_1_ to form a polymer(P_1_) having a shorter chain length.

### Morphological analysis

**Figure 5:**
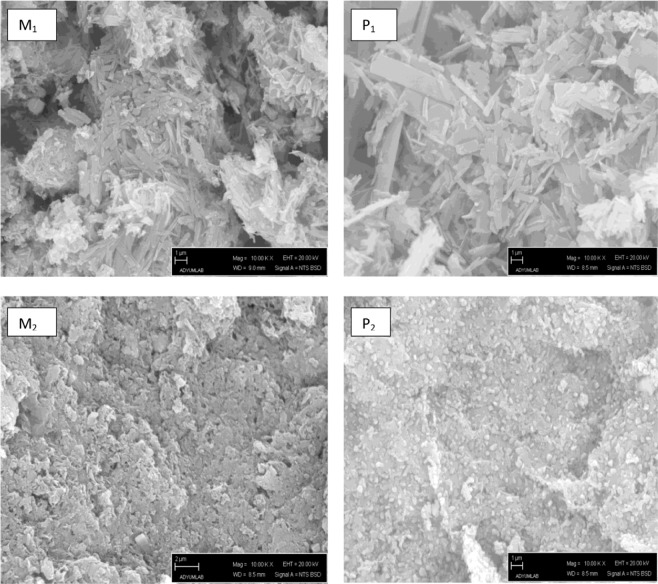
Scanning electron micrographs (SEM) image of azo monomers M_1_, M_2_ and polymer P_1_, P_2_ under 10.00 kV.

By taking the SEM images of the synthesized monomers and polymers in different sizes, the information about their subgrain structures was obtained. The most obvious difference in the SEM images (Fig. 5) is that the polymer granule size is larger than the monomer granule size. According to the obtained SEM images, both monomers and polymers have a laminar structure. However, in the monomer, the layers are in the shape of smaller particles, and there are porous structures between the granules. Moreover, it is also observed that the laminar structure of the polymer is more regular than the monomer. On the surface of allsynthesized compounds, there are granules which are independent of the matrix structure. Unlike polymer, Monomer granules are observed in the form of an agglomerate.

### UV-vis absorption spectra and solvent effects

The electronic transitions of azo dyes strongly depend on the nature of the media. For this purpose, the electronic absorption spectra of the synthesized azo monomer and polymer were measured in the range of 200-700 nm in six solvents (polar protic, polar aprotic and aprotic) with different polarity and hydrogen bonding property at room temperature.

**Figure 6:**
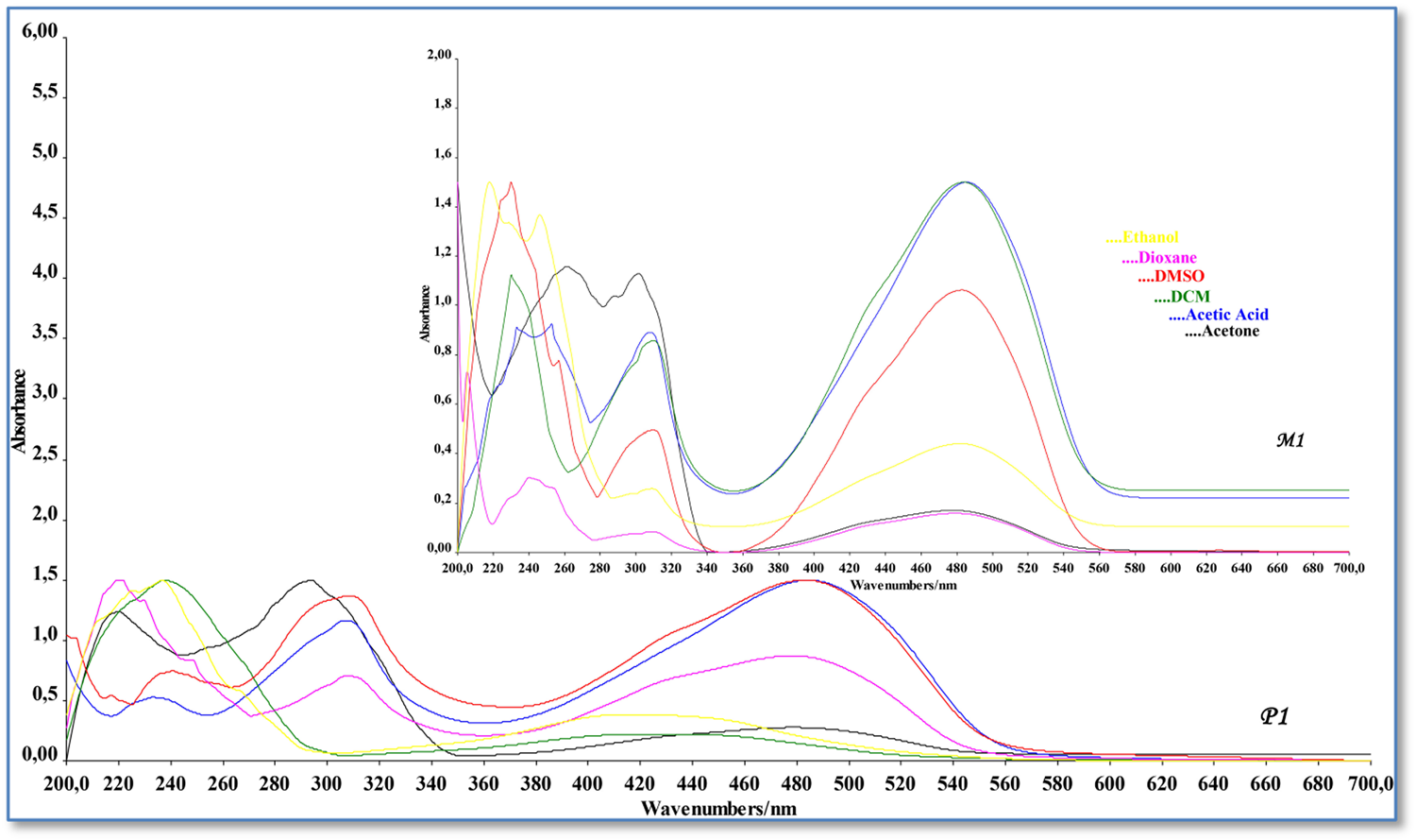
UV-Vis spectra of azo monomer M_1_ and polymer P_1_ in DMSO, DMC, acetone, ethanol, dioxsane, acetic acid.

The UV-vis absorption of M_1_ in six solvents are shown in Fig. 6. The first and second bands located at 205–261 nm were assigned to the moderated energy π → π* transition of aromatic rings. Although the polarization of the solvent was changed, while the first band was formed in the similar region, differences occurred in the second band. The third band was measured in the range of 301–309 nm due to π → π* transition involving the π-electrons of azo group. Absorption intensity of π → π* transition increased due to the hyperchromic effect (DMSO > Ethanol = Dioxane = Acetic Acid > DCM > Acetone). The π → π* transition in DMSO was observed at a higher wavelength (batochromic effect) with higher absorption intensity than other solvents (Table 1). The reason for this was that the dipole moment of DMSO affected the dipole moment of M_1_. In polar solvents, whereas π is an orbital that can be less polarized, π* is an orbital that can be polarized easily or affected, and its energy level decreases more than π orbital. As a result, the π → π* transition energy decreases and the absorption of this transition shifts to longer wavelengths (301–309 nm). The fourth bands were observed in the range of 477–485 nm due to charge-transfer complex transition(CTC). A charge-transfer complex is the interaction based on the charge transfer or the transfer of a pair of electrons from the donor to the acceptor. Similarly, in the complex formation by the interaction of two different molecular or ionic structures, the rearrangement of the electronic charge in the complex can occur by stimulating an electron with a photon in the complex^39^. The charge-transfer complex consists of the lowest energy hollow molecular orbital (LUMO) of the acceptor and the highest energy filled molecular orbital (HOMO) of the donor^40^. When these two orbits are in proper orientations, load transfer from the donor to the acceptor takes place. This is a stability enhancing interaction.

**Table 1:** Electronic absorption data of the synthesized azo monomer(M_1_).

Solvent	A		B		C		D		
	λ_max_	ε_max_^a^	λ_max_	ε_max_^a^	λ_max_	ε_max_^a^	λ_max_	ε_max_^a^	E_CTC_^b^
Ethanol	—	—	246	75.128	308	9.207	481	20.014	2.583
Dioxane	205	87.736	240	36.194	308	9.317	479	18.342	2.594
DMSO	—	—	230	11.551	309	8.105	483	16.688	2.573
DCM	230	26.74	256	13.445	307	8.394	482	18.534	2.578
Acetone	—	—	261	121.446	301	118.258	477	12.594	2.605
Acetic acid	233	11.043	252	11.233	308	10.735	485	20.430	2.562

A, B, C, D (Absorption band), λ_max_(Absorption maxima), ^a^Molar absorptivity coefficient (mol^−1^ l cm^−1^), ^b^Charge transfer conplex energy(x10^4^).

The CTC transition was observed in all solvents used for M_1_. In the electronic absorption spectra, it was observed that fourth bands (CTC) were broader than the first, second and third bands because the nature of the band was sensitive to the type of the solvent. The absorption of λ_max_ of CTC transition in acetic acid was higher than other solvents used. The reason for this was that the polarization of the acetic acid solvent had more effect on the n orbital of M_1_.

The UV-vis absorption spectra of M_2_ showed four absorption bands in the visible region (Table 2). The obtained results showed that although lightly positive solvatochromism in uv-vis range was evident, the absorption spectra data of M_2_ did not change significantly in all the used solvents and the absorption maxima did not relate with the polarity of the solvent. The little absorption changes in the absorption spectra occurred because of intramolecular hydrogen bonding in the azo compounds^41^. The π → π* transition of aromatic rings were observed in the range of 220–270 nm. The first and third transition bands did not occur in DMSO and DCM solvents. Also, second transition bands were not observed in the acetic acid solvent. The CTC transition data in all solvents used occurred independently from solvent polarity. The highest value of CTC transition occurred in ethanol (λ_max_ is 481 nm). The highest value of CTC transition occurred in ethanol while the lowest value in dioxane observed.

**Table 2:** Electronic absorption data of the synthesized azo monomer(M_2_).

Solvent	A		B		C		D		
	λ_max_	ε_max_^a^	λ_max_	ε_max_^a^	λ_max_	ε_max_^a^	λ_max_	ε_max_^a^	E_CTC_^b^
Ethanol	229	64.438	239	65.002	309	8.178	481	20.100	2.583
Dioxane	220	27.146	246	11.728	—	—	414	22.388	3.001
DMSO	—	—	240	4.884	—	—	416	22.068	2.987
DCM	—	—	239	124.666	—	—	433	15.924	2.870
Acetone	238	79.214	270	104.384	311	68.142	424	17.932	2.931
Acetic acid	223	6.538	—	—	272	1.655	417	19.960	2.980

A,B,C,D (Absorption band), λ_max_(Absorption maxima), ^a^Molar absorptivity coefficient (mol^−1^ l cm^−1^), ^b^Charge transfer complex energy(x10^4^).

**Figure 7:**
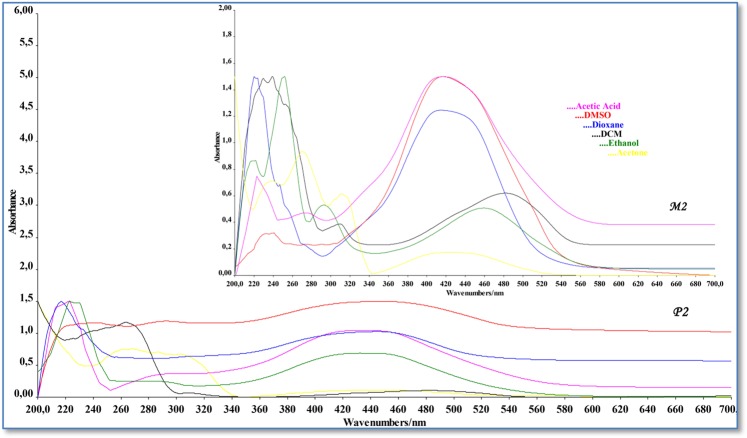
UV-Vis spectra of azo monomer M_2_ and polymer P_2_ in DMSO, DMC, acetone, ethanol, dioxsane, acetic acid.

Using six solvents with different properties as in M_1_, the UV–vis absorption spectra of P_1_ was recorded over the range of λ_max_ between 200 and 700 nm (Fig. 7). The most obvious difference seen in the P_1_ spectra was that some of the π → π* transitions absorption bands seen in M_1_ spectra did not occur in the P_1_ spectra. For the polymer, this situation was observed in all solvents. Because of the different interactions in the resonance system and the solvents, while the charge-transfer complex transition(CTC) of P_1_ was shifting red in the acetone, ethanol and DCM solvents (λ_max_ is 485 nm in acetone, 489 nm in ethanol and 491 nm in DCM), it was shifting blue in the acetic acid solvent (λ_max_ is 481 nm) according to the monomers it had (Table 3). The P_2_ (Fig. 7) showed four absorption maxima, first and second bands in the ultraviolet region in the range 218–257 nm and third band in the visible region due to π → π* transition of azo linkage N=N of dyes (291–306 nm). Also, the first band that was occurred owing to π → π* transition did not occur in DMSO, DCM and ethanol solvents. CTC transition was observed in all solvents used for P_2_. In the UV–vis absorption spectra of P_2_, The red shift was formed the CTC bands in solvents except for the acetic acid solvent (Table 4).

**Table 3:** Electronic absorption data of the synthesized azo polymer(P_1_).

Solvent	A		B		C		D		
	λ_max_	ε_max_^a^	λ_max_	ε_max_^a^	λ_max_	ε_max_^a^	λ_max_	ε_max_^a^	E_CTC_^b^
Ethanol	225	11.036	—	—	307	106.682	489	13.110	2.541
Dioxane	221	18.354	—	—	309	8.320	479	13.480	2.594
DMSO	217	948	240	3.108	308	9.754	484	12.892	2.567
DCM	—	—	264	145.528	308	9.126	491	16.294	2.531
Acetone	220	64.834	—	—	293	106.220	485	13.266	2.562
Acetic acid	—	—	233	4.092	307	10.916	481	15.070	2.583

A, B, C, D (Absorption band), λ_max_(Absorption maxima), ^a^Molar absorptivity coefficient (mol^−1^ l cm^−1^), ^b^Charge transfer complex energy(x10^4^).

**Table 4:** Electronic absorption data of the synthesized azo polymer(P_2_).

Solvent	A		B		C		D		
	λ_max_	ε_max_^a^	λ_max_	ε_max_^a^	λ_max_	ε_max_^a^	λ_max_	ε_max_^a^	E_CTC_^b^
Ethanol	—	—	238	23.632	291	7.142	439	21.370	2.831
Dioxane	223	50.404	251	50.024	303	23.336	444	59.764	2.799
DMSO	—	—	247	43.298	292	25.572	451	68.794	2.755
DCM	—	—	257	121.660	306	4.382	446	18.194	2.786
Acetone	219	87.750	253	109.244	303	99.390	447	31.722	2.780
Acetic acid	218	21.876	253	25.830	300	13.962	437	57.170	2.843

A, B, C, D (Absorption band), λ_max_(Absorption maxima), ^a^Molar absorptivity coefficient (mol^−1^ l cm^−1^), ^b^Charge transfer complex energy(x10^4^).

Charge-transfer complex band originates from the formation of a link between the -OH group and the N=N group behaving as an acceptor, and from the lone-pairs in the molecule. In this interaction, the -OH group behaves as a donor^42^. This assumption can be confirmed by calculating the energy charge transfer (E_CTC_) from λ_max_ by using the equation below:

$${{\rm{E}}}_{{\rm{CTC}}}=1242.6/({{\rm{\lambda }}}_{{\rm{\max }}}({\rm{nm}}))({\rm{eV}})$$

When we examined the E_CTC_ values of the compounds, while the highest value formed in acetone solvent for M_1_, and in dioxane solvent for M_2_. M_1_ E_CTC_ values are lower compared with M_2_. The reason for this is the fact that the bands of M_1_ in the visible region formed at higher wavelengths. In polymers, the highest value for P_1_ formed in dioxane and in acetic acid for P_2_.

Molar absorption coefficients(ε) of azo monomers and polymers are given in Tables 1–4. The data obtained show how much light the compounds absorb in polar protic, polar aprotic and aprotic solutions. Since UV-vis light consists of photons having their own energies (or wavelengths), one photon was absorbed to a greater degree than the other depending on the compound being analyzed; that is, light absorbed at certain wavelengths depending on the characteristics of the material. Therefore, the molar absorption values of the synthesized compounds are directly proportional to the degree of light absorption at a given wavelength. Azo grubu (-N=N-) yüksek molar absorbans değerlerine sahip olan kromofor gruplardır. Since molar absorbance is directly proportional to the molar absorbance coefficient (A = ε.c.l), the molar absorption coefficient of azo compounds is high too. This property is seen in synthesized azo compounds. It is well known that λmax values tend to be related to the strength of the electronic power in the benzenoid system^43^. Since, the electronic transition in these compounds takes place from the donor group to the azo group, In particular, binding of the substituents to the azo group from the ortho or para positions increases the conjugation which leads to absorption at longer wavelengths^44^. The molar absorption coefficient of the synthesized compounds varied according to the polarity of the solution in which they are present. The molar absorption coefficient values of M_1_, M_2_, P_1_ and P_2_ are 12.594–20.430 mol^−1^1 cm ^−1^, 15.924–22.388 mol^−1^1 cm ^−1^, 12.892–15.070 mol^−1^1 cm ^−1^, and 18.194–57.170 mol^−1^1 cm ^−1^, respectively. According to the data obtained, the highest molar absorption coefficient belongs to P_2_ (57.170 mol^−1^1 cm ^−1^ in Acetic acid). The conjugation in the molecular structure of P_2_ increased due to having a long chain structure. Therefor, Increased conjugation and high solvent interaction with polar protic acetic acid have increased the light absorption of P_2_.

### Thermal degradation behavior study

**Figure 8:**
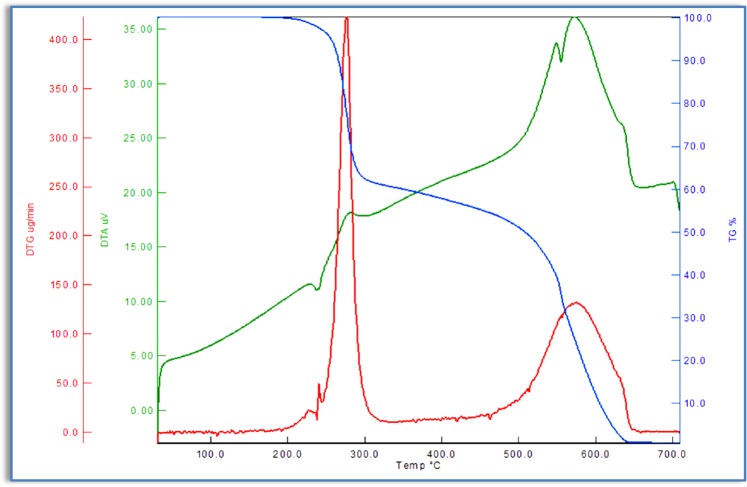
TG, DTG and DTA curves of azo monomer M_1_.

**Figure 9:**
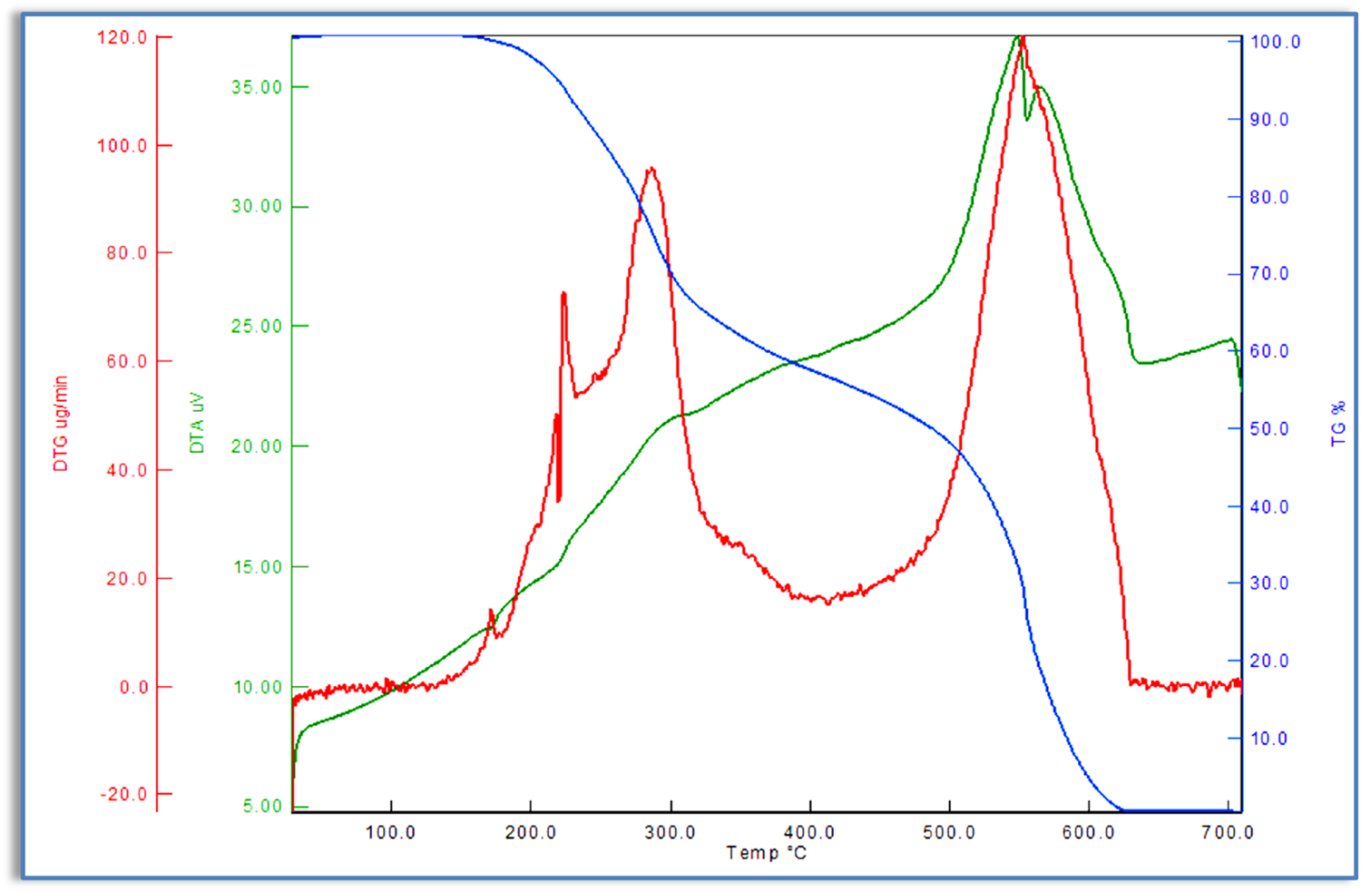
TG, DTG and DTA curves of azo monomer M_2_.

In order to determine the thermal stability of azo monomers and polymers, thermogravimetric analysis (TGA/DTG/DTA) were carried out under N_2_ atmosphere within the temperature range of 30–700 °C. The temperature ranges and mass losses percentages of the decomposition reaction were given in Tables 5 and 6. A general decomposition, in which the azo monomers were in two steps as shown in Fig. 8 was obtained. The TG curve of M_1_ shows two stages of mass losses at temperature ranges from 198 to 648 °C. These stages contain mass losses of about 100% at the given temperature ranges. The first stage occurs with a weight loss of 61.19% within the temperature range of 198–317 °C. It is probably due tothe thermal decomposition and elimination of chlorine atom and benzene bonded to the ether. The DTA curve of M_1_ shows an exothermic sharp peak with its maximum value at 276 °C. The second stage, in the temperature range of 483–648 °C, is observed with a mass loss of 38.08%, corresponding to the elimination of the organic residue. The exothermic DTA peak at 575 °C supported this decomposition. The thermogravimetric analysis result of M_2_ was shown in Fig. 9. The absence of any weight loss up to 100 °C indicates that there are no crystal water molecules in the structure of M_2_. The TGA curve of M_2_ showed two stages of thermal decomposition. The first stage occurred within the temperature range 150–415 °C, with a weight loss of 56.36% due to the loss of benzene unit from the azo monomer. The second stage, in the temperature range 488–636 °C, was observed with a mass loss of 43.16%, corresponding to the elimination of the organic residue from the remaining azo monomer.

**Table 5:** Thermal stability and thermal degradation data of synthesızed monomers and polymers.

Compounds	TG						
	T_i_(^o^C)	T_25_(^o^C)	T_50_(^o^C)	T_75_(^o^C)	T_f_(^o^C)	T_max_(^o^C)	Residue(%)
M_1_	198	278	508	575	648	276–575	0.73
M_2_	150	287	486	556	636	287–552	0.43
P_1_	171	333	529	568	650	269–557	1.06
P_2_	147	298	488	565	641	288–517–604	0.94

T_i_: İnitial decomposition temperature, T_25_: Temperature for 25% weight loss, T_50_: Temperature for 50% weight loss,

T_75_: Temperature for 75% weight loss, T_f:_ End of decomposition.

**Table 6:** TG-DTA characteristic parameters of synthesized monomers and polymers at heating rate of 10 °C/min.

Compounds	Stage I			Stage II			Stage III			W_tl_ (%)
	T_r_ (^o^C)	W_l_ (%)	DTG_max_ (^o^C)	T_r_ (^o^C)	W_l_ (%)	DTG_max_ (^o^C)	T_r_ (^o^C)	W_l_ (%)	DTG_max_ (^o^C)	
M_1_	198–317	61.19	276	483–648	38.08	575	—	—	—	99.27
M_2_	150–415	56.36	287	488–636	43.16	552	—	—	—	99.52
P_1_	171–348	74.17	269	398–650	24.77	557	—	—	—	98.94
P_2_	147–339	65.22	288	478–545	30.74	517	556–641	3.10	604	99.06

T_r_: Mass loss temperature ranges, W_l_: Percentage of lost mass of the sample in the temperature range, W_tl_: Percentage of total lost mass.

**Figure 10:**
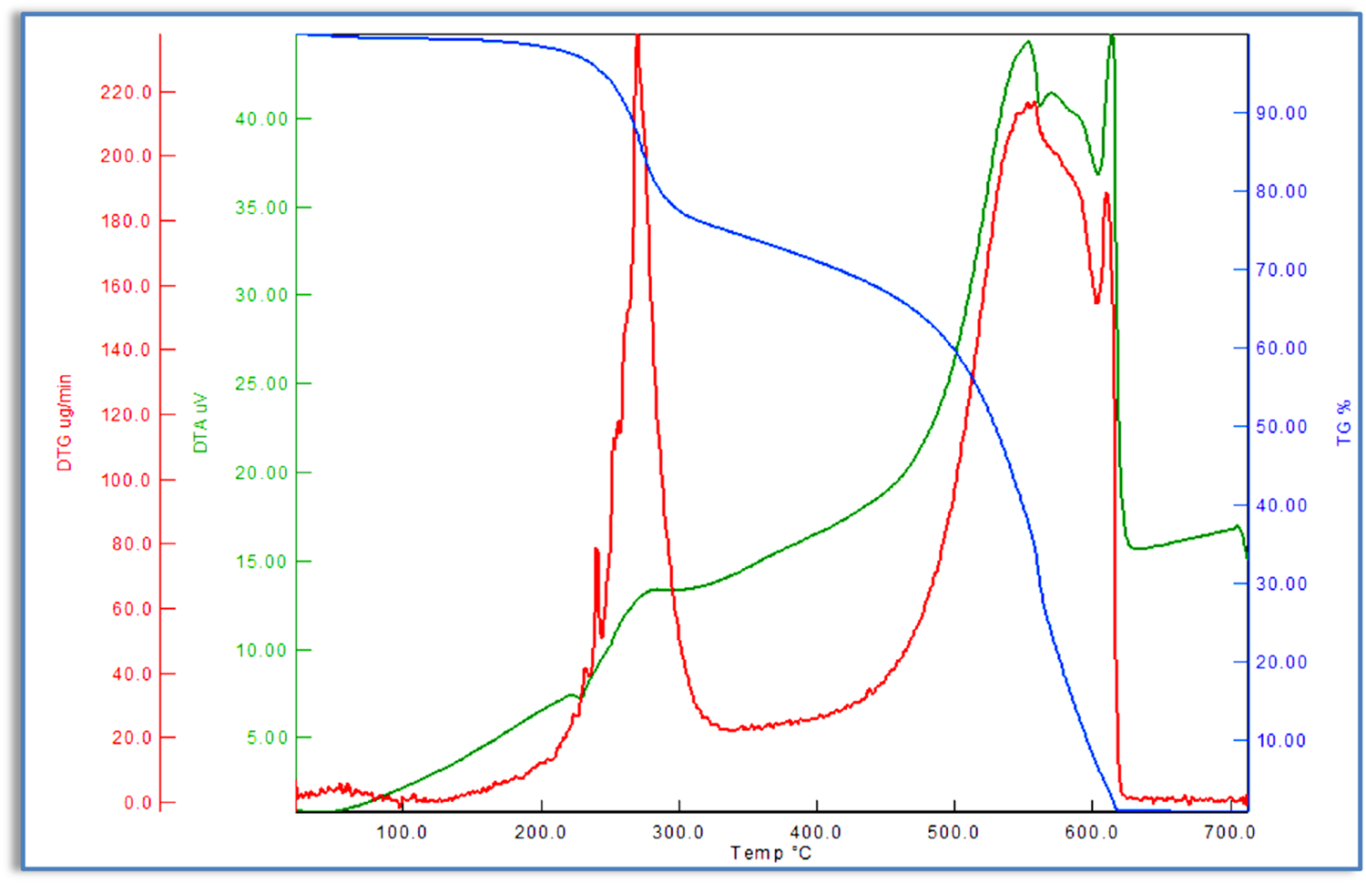
TG, DTG and DTA curves of azo polymer P_1_.

Figure 10 shows weight loss curves for the studied P_1_. The mass loss and degradation speed of polymer were illustrated in Tables 5 and 6 From these data, it is possible to precisely determine the thermal stability. Unlike M_1_, the degradation kinetics of polymer is due to the different chemical structure. It was observed from the TG curves of P_1_ that the weight losses occurred in two steps within the temperature range of 171–650 °C. In the first step with a minimum value of DTG at 269 °C, 74.17% weight loss was observed in the temperature range of 171–348 °C. In the second step, 24.77% weight loss was recorded in the temperature range of 398–650 °C. The peak with a maximum value at 557 °C in the DTG curve corresponds to this degradation step. Both stages are exothermic decompositions according to DTA curve.

**Figure 11:**
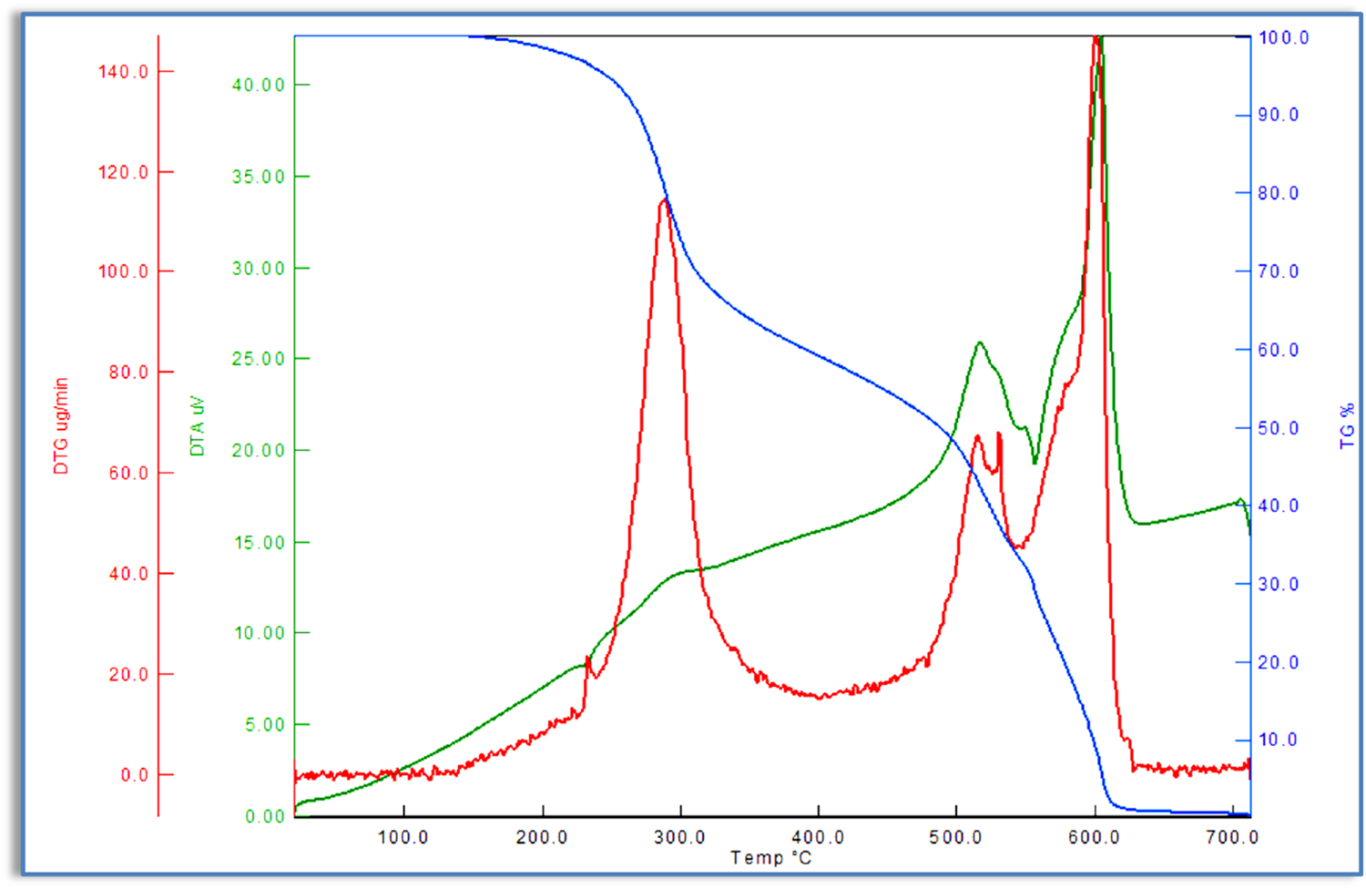
TG, DTG and DTA curves of azo polymer P_2_.

The thermograms of the P_2_ show three steps of decomposition within the temperature range of 147–641 °C (Fig. 11). The small amount of mass loss up to 100 °C was caused by water molecules in the structure of the P_2_. The first step occurred within the temperature range of 147–339 °C with mass losses of 65.22%. Then, the second step thermal decomposition curve started within a temperature range of 478–545 °C which corresponds to a mass loss of 30.74% associated with two DTA peak at 517 °C. Finally, at the third step (556–641 °C) showed one endothermic DTG peak at 604 °C with and this corresponds to the release of the remaining 0.94%.

### Kinetic and thermodynamic analysis

Different equations were suggested to determine the kinetic parametersin thermal degradation reactions. Data obtained from TG curve are used to form these equations. Geometric structure of the TG curve provides us information on thermal stability of by- and end-products of degraded substances and allows us to obtain physicochemical data of degradation composition at the same time. In calculating the kinetic parameters of thermal degradation curves, kinetic analysis methods, which start with the Arrhenius equations, are used. The basic equation was given below.

1 $$\frac{d\alpha }{dt}=k(T)f(\alpha )$$

where the term dα/dt denotes the reaction rate, k is the reaction constant, α is the conversion degree, T is the process temperature and f(a) is the reaction model, a function depending on the actual reaction mechanism.

2 $$\alpha =\frac{Wo-Wt}{Wo-Wf}$$

The conversion degree α is defined as: where W_o_ is the initial mass of sample, W_f_ is the final mass of sample and W_t_ is the mass of the sample at a time.

3 $$k(T)=Aexp(-\frac{Ea}{RT})$$

In which A is pre-exponential factor (min^−1^), Ea is activation energy (kJ/mol),T is reaction temperature (K) and R is universal gas constant (0.008314 kJ/mol K). For non-isothermal experiments conducting at constant heating rate β = dT/dt, the decomposition rate equation can be simplified and rearranged to the right part of Eq. 4.

4 $$\frac{d\alpha }{dT}=\frac{A}{\beta }exp(\frac{Ea}{RT})f(\alpha )$$

The integral form of Eq. 4 can be written as follows:

5 $$f({\rm{\alpha }})={\int }_{0}^{\alpha }\frac{d\alpha }{f(\alpha )}=\frac{A}{\beta }{\int }_{To}^{T}exp(\frac{Ea}{RT})dT$$

Equation 5 are the fundamental expressions of analytical methods to calculate kinetic parameters on the basis of TGA data^45^. This equation was developed by many researchers, and a number of methods for different situations were proposed. In this study, Coats-Redfern, Broido and Horowitz-Metzger methods were employed to calculate the kinetic parameters ((activation energy (Ea), enthalpy (∆H), entropy (∆S), and Gibbs free energy change of the decomposition (∆G)).

Coats-Redfern is one of the non-isothermal model-fitting methods. This kinetic model depends on the integral form of reaction models and uses asymptotic series expansion. The main equation for Coats-Redfern method is given below

6 $$Log[\frac{g(\alpha )}{{T}^{2}}]=Log[\frac{AR}{\beta E}(1-\frac{2RT}{E})]-\frac{E}{2.303RT}$$

According to the Coats-Redfern method, the activation energies Ea can be calculated from the slope of the linear fitted line between[log (−g(α)/T^2^)] and 1/T. Also, A was determined from the intercept of the line. To calculate the kinetic parameters, thermal degradation reaction mechanism is assumed first order (n = 1).

Broido

Broido developed the statement below for only first-order reactions using different assumptions to realize the integration of Eq. 4.

7 $$ln[g(\alpha )]=ln[\frac{A}{\beta }(\frac{R}{E}){T}_{m}^{2}]-\frac{E}{RT}$$

In the plot of $ln\lceil g(\alpha )\rceil$ versus 1/T, the E values can be obtained from their slopes, respectively. The intercept from the plot is $ln[\frac{A}{\beta }(\frac{R}{E}){T}_{m}^{2}]$ and can be calculated the A value.

**Figure 12:**
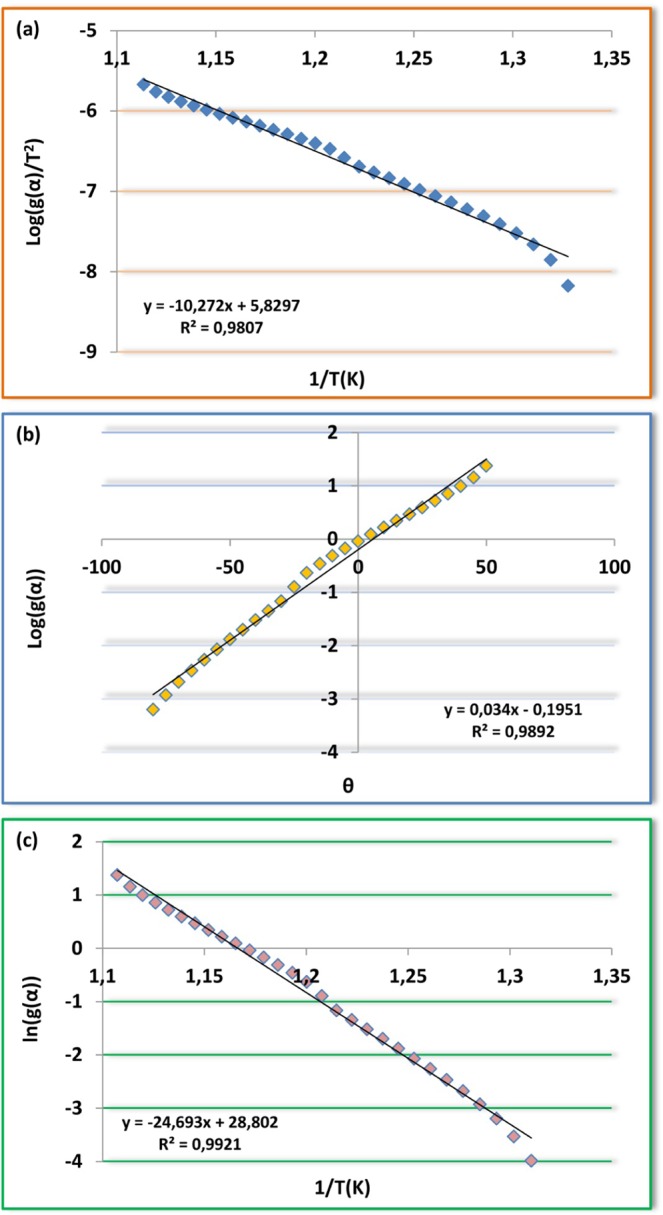
Plots of M_1_ (**a**) Coats-Redfern, (**b**) Horowitz-Metzger, (**c**) Broido.

**Figure 13:**
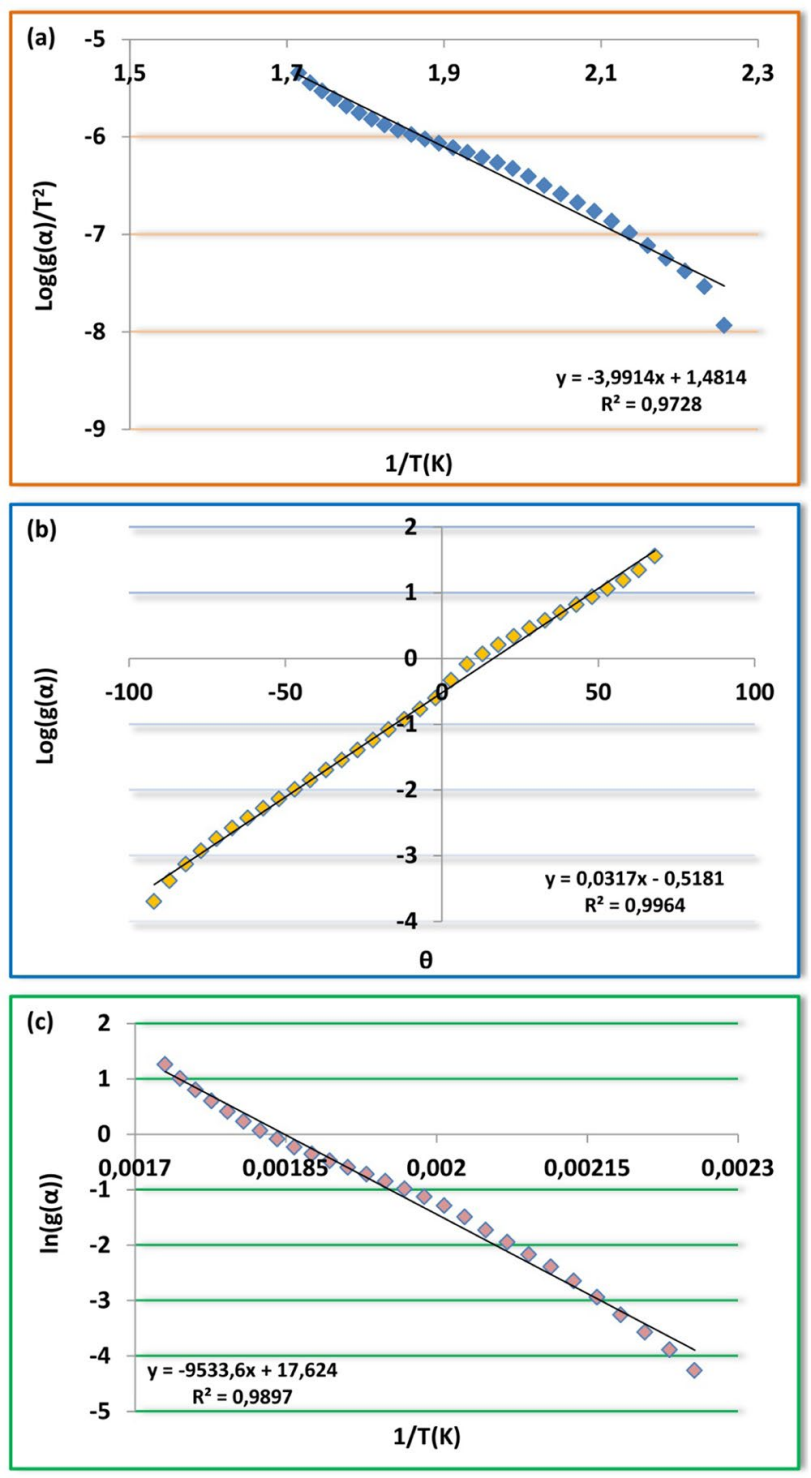
Plots of M_2_ (**a**) Coats-Redfern, (**b**) Horowitz-Metzger, (**c**) Broido.

**Figure 14:**
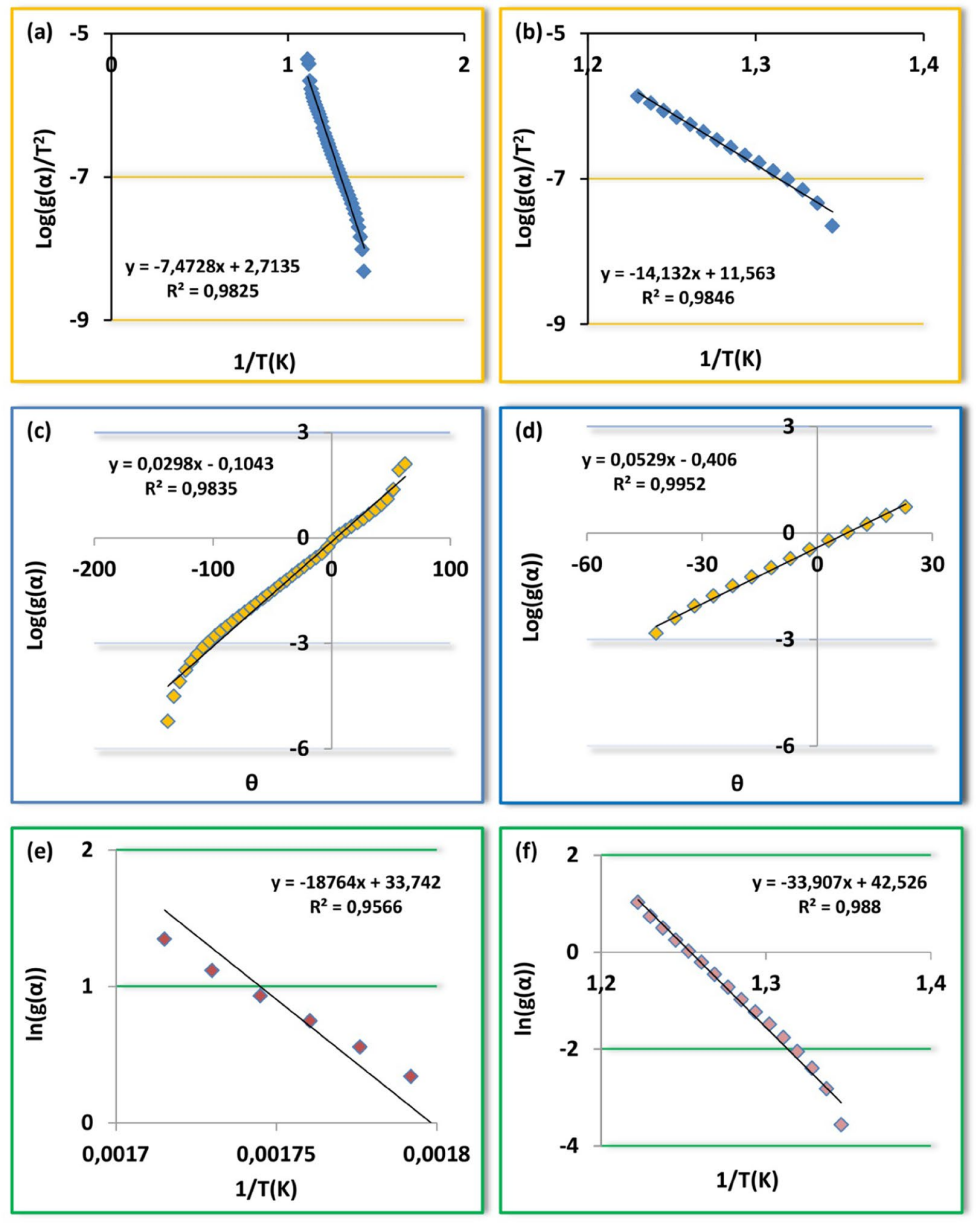
Plots of polymers (**a**) Coats-Redfern; P_1_, (**b**) Coats-Redfern; P_2_, (**c**) Horowitz-Metzger; P_1_, (**d**) Horowitz-Metzger; P_2_, (**e**) Broido; P_1_, (**f**) Broido; P_2_.

Horowitz-Metzger

8 $$Log[g(\alpha )]=\frac{E\theta }{2.303R{T}_{m}^{2}}$$

where θ = T − Ts, Ts is the DTG peak temperature, T is the temperature corresponding to weight loss Wt. A plot of $Log[g(\alpha )]$ versus θ should give a straight line whose slope is $E/R{T}_{m}^{2}$ (𝛼) = −ln(1 − 𝛼) indicates random nucleation model for the degradation of the material (Figs. 12–14). The preexponential factor (*A*) is calculated by using the Eq. 9.

9 $$A=\frac{E}{R{T}_{m}^{2}}\beta exp(\frac{E}{R{T}_{m}})$$

Here, β is the heating rate.

Thermodynamic parameters such as changes in Enthalpy(∆H), Gibbs free energy(∆G) and the Entropy of activation (ΔS) in J K^−1^ mol^−1^, was calculated using the equations:

10 $$\Delta H=E-R{T}_{m}$$

11 $$\Delta G=\Delta H-\Delta S{T}_{m}$$

12 $$\Delta S=2.303{\rm{R}}(\frac{Ah}{k{T}_{m}})$$

where T_m_ is the DTG peak temperature, R is universal gas constant, k is the Boltzmann constant and h is Planck’s constant.

The kinetic parameters of monomers and azo polymers which were obtained by using Coats-Redfern, Broido, and Horowitz-Metzger methods were given in Tables 7–10 respectively. Enthalpy (∆H), states the change in energy of a system that occurs during a chemical reaction under constant pressure. Enthalpy change is an internal state function and does not depend on the path. When the enthalpy values of the azo monomers are compared, it is seen that the highest value took place at the 1^st^ phase of degradation of M_1_ (Coats-Redfern ∆H: 313.08 kjmol^−1^, Broido ∆H: 257.04 kjmol^−1^, Horowitz-Metzger ∆H: 197.75 kjmol^−1^). The lowest enthalpy value formed at the 1^st^ phase of M_2_. Also, the total enthalpy change that occurs during thermal degradation formed the lowest at M_2_ between all the compounds synthesized. This indicates that the internal energy of M_2_ is lower than those of the other compounds synthesized. In azo polymers, it is seen that the enthalpy values of P_2_ are higher compared with those of P_1_. The highest enthalpy change occurred especially at the 3^rd^ phase of P_2_ (Coats-Redfern ∆H: 338.85 kjmol^−1^, Broido ∆H: 401.95 kjmol^−1^, Horowitz-Metzger ∆H: 326.60 kjmol^−1^). All the enthalpy changes that occur during thermal degradations are positive, and this indicates that the reactions occurred are endothermic. This is also proved with the obtained DTA and DTG curves.

**Table 7:** Activation energy (Broido, Coats-Redfern and Horowitz-Metzger merhods) and fractional conversion of synthesızed monomers and polymer.

Compounds	Stages	Broido	Coats-Redfern	Horowitz-Metzger	
		Ea(kJmol^−1^)	Ea(kJmol^−1^)	Ea(kJmol^−1^)	α
M_1_	Stg-1st	317.65	197.75	261.04	0.1146
	Stg-2nd	205.29	191.89	203.27	0.7641
M_2_	Stg-1st	83.86	74.01	90.73	0.2488
	Stg-2nd	174.72	161.46	180.03	0.7007
P_1_	Stg-1st	156.00	164.02	146.54	0.1231
	Stg-2nd	151.65	138.45	170.67	0.6478
P_2_	Stg-1st	199.78	187.94	169.55	0.1915
	Stg-2nd	281.90	266.28	267.58	0.5797
	Stg-3rd	346.14	326.60	409.25	0.9421

**Table 8:** Kinetic parameters of synthesızed monomers and polymers at heating rate of 10 °C/min. using Broido equation.

Compounds	Stages	A(s^−1^)	∆S(Jmol^−1^K^−1^)	∆H(kjmol^−1^)	∆G(kjmol^−1^)	K(s^−1^)	R^2^
M_1_	Stg-1st	1.10.10^30^	−249.20	313.08	449.90	0.206	0.971
	Stg-2nd	1.10.10^12^	−61.33	198.24	250.25	0.255	0.992
M_2_	Stg-1st	3.44.10^4^	−163.33	79.21	170.68	0.518.10^−3^	0.989
	Stg-2nd	2.06.10^10^	−49.02	168.09	208.53	0.178	0.996
P_1_	Stg-1st	2.88.10^14^	−26.92	155.99	139.78	0.269	0.956
	Stg-2nd	2.87.10^11^	−53.07	144.71	195.44	0.820	0.985
P_2_	Stg-1st	1.24.10^15^	−19.63	195.12	184.10	0.311.10^−3^	0.958
	Stg-2nd	1.56.10^15^	−37.84	275.33	245.43	0.361.10^−3^	0.988
	Stg-3rd	2.94.10^17^	−99.68	338.85	251.42	0.619.10^−4^	0.963

**Table 9:** Kinetic parameters of synthesızed monomers and polymers at heating rate of 10 °C/min. using Horowitz-Metzger equation.

Compounds	Stages	A(s^−1^)	∆S(Jmol^−1^K^−1^)	∆H(kjmol^−1^)	∆G(kjmol^−1^)	K(s^−1^)	R^2^
M_1_	Stg-1st	8.08.10^24^	245.90	257.04	122.04	0.456	0.969
	Stg-2nd	1.12.10^12^	−22.88	196.22	222.68	0.338	0.989
M_2_	Stg-1st	1.00.10^8^	−96.99	86.07	137.39	0.346	0.981
	Stg-2nd	7.91.10^9^	−44.74	173.17	210.08	0.031	0.996
P_1_	Stg-1st	7.94.10^13^	16.22	142.03	133.24	0.604	0.946
	Stg-2nd	1.63.10^10^	−57.89	163.77	211.83	0.296	0.983
P_2_	Stg-1st	3.97.10^15^	−48.46	164.89	137.70	0.649	0.973
	Stg-2nd	4.38.10^17^	−84.84	261.09	186.68	0.898	0.995
	Stg-3rd	1.51.10^24^	−208.60	401.95	219.01	0.642	0.993

**Table 10:** Kinetic parameters of synthesızed monomers and polymers at heating rate of 10 °C/min. using Coats-Redfern equation.

Compounds	Stages	A(s^−1^)	∆S(Jmol^−1^K^−1^)	∆H(kjmol^−1^)	∆G(kjmol^−1^)	K(s^−1^)	R^2^
M_1_	Stg-1st	4.46.10^18^	107.05	197.75	139.94	0.199	0.958
	Stg-2nd	1.59.10^11^	−58.31	191.89	264.24	0.123	0.980
M_2_	Stg-1st	2.96.10^6^	−126.28	74.01	144.72	0.223	0.972
	Stg-2nd	2.63.10^9^	−73.04	161.46	221.72	0.033	0.990
P_1_	Stg-1st	1.07.10^9^	−77.03	164.02	205.77	0.168.10^-6^	0.932
	Stg-2nd	8.89.10^7^	−101.26	138.45	222.50	0.088	0.982
P_2_	Stg-1st	6.27.10^16^	71.41	187.94	147.88	0.119	0.953
	Stg-2nd	1.20.10^17^	73.39	266.28	207.85	0.005	0.984
	Stg-3rd	1.65.10^19^	144.03	326.60	200.29	0.292	0.959

Entropy (∆S), is an indicator of a system’s disorder. As in the changes in enthalpy, changes in entropy depend only on the first and last state of a system. It does not depend on the paths on which the reaction takes. An increase in entropy is an indicator of the reaction occurring spontaneously. When the entropy values of thermal degradation phases of azo monomers that were obtained in the methods studied were examined, it was seen that the first degradation phases are higher compared with the second degradation phases. This increase in entropy shows that the first phase occurred more easily than the second phase. In azo polymers, different from the azo monomers, higher entropy values occurred at the second phase for P_1_ and third phase for P_2_. This is because azo polymers consisted of polymer chains that have different molecular weights and chain lengths converge to each other with the effect of thermal decomposition as the temperature rises.

Gibbs energy, which represents the useful work that a system produces, is a state function and can be used to evaluate if a change in state will occur spontaneously or not. That the ΔG° value is negative or positive indicates that whether or nota reaction occursspontaneously. If the ΔG° value is negative, it shows that the reaction is spontaneous towards the products (exergonic); and if it is positive, then it shows that the reaction is non-spontaneous towards the products and spontaneous towards the reactants (endergonic) (ΔG° < 0 exergonic, ΔG° > 0 endergonic). The obtained data show that the azo monomers and the azo polymers have positive ΔG° value and an increase occurs in ΔG° value together with an increase in temperature. This is a proof for the fact that the synthesized compounds perform the thermal degradation reactions in an endergonic way. An increase in ΔG° values indicates that the endergonic state increases against thermal degradation together with an increase in temperature.

## Conclusion

In the first stage of the study, the novel two azo monomers, 3-((5-chloro-2-phenoxyphenyl)diazenyl)naphthalene-2,7-diol and 4-((5-chloro-2-phenoxyphenyl)diazenyl)benzene-1,3-diol, were prepared by coupling the reaction of 2,7-dihydroxynaphthalene, 1,3-benzenediol and 2-amino-4-chlorophenyl phenyl ether. Then, the synthesized azo compounds were polymerized by oxidative polycondensation reaction. The prepared azo monomers and polymers were characterized as ^1^H-NMR, FT-IR, UV-vis and GPC spectra data. Theoretical data of azo monomers were compared with experimental results. According to the obtained results, generally FT-IR and UV-vis results are compatible with each other. According to the results, while monomers showed a positive solvatochromic behavior in some solvents, it showed a negative solvatochromic behavior in some other solvents. This indicates that the absorption features of monomers not only depend on the polarity of the solvent. According to absorption data, the polymers also showed similar absorption features to those of monomers. The reason for this was that dipolarity/polarizability interactions between compounds and solvents were different. With respect to the results of the thermal analysis, it was observed that azo monomers and polymers had different resistance against heat. According to thermal initial degradation temperature of polymers and monomers, there is no significant difference among the synthesized compounds. While the initial decomposition temperature of the polymers (P_1_T_İ_: 171 °C, P_2_T_İ_: 147 °C) were lower than the monomer (M_1_T_İ_: 198 °C, M_2_T_İ_: 150 °C), half-life (P_1_T_50_: 529 °C, P_2_T_50_: 488 °C) and final decomposition temperatures (P_1_T_f_: 650 °C, P_2_T_f_: 641 °C) were higher than the monomers (M_1_T_50_: 508 °C, M_2_T_50_: 486 °C, M_1_T_f_: 648 °C, M_2_T_f_: 636 °C). Even though the first decomposition temperature of the polymers is lower than the monomer, the temperature resistance of the polymers is better than the monomers according to their half-life and final decomposition temperatures. The activation energy (E_a_) determined by Coats-Redfern, Broido, and Horowitz-Metzger methods allowed for observation the difference between the degradation intervals of compaunds. According to the data obtained, when the activation energy of the synthesized compounds are compared, it is seen that the highest value took place at the 3^rd^ phase of degradation of P_1_(Coats-Redfern E_a_: 346.14 kjmol^−1^, Broido E_a_: 326.60 kjmol^−1^, Horowitz-Metzger E_a_: 409.25 kjmol^−1^). The results obtained from this study showed that the thermal properties of azo monomers are differentiated by polymerization and more stable compounds was obtained.
